# Therapeutic opportunities in Ewing sarcoma: EWS-FLI inhibition *via* LSD1 targeting

**DOI:** 10.18632/oncotarget.7124

**Published:** 2016-02-02

**Authors:** Emily R. Theisen, Kathleen I. Pishas, Ranajeet S. Saund, Stephen L. Lessnick

**Affiliations:** ^1^ Center for Childhood Cancer and Blood Disorders, The Research Institute at Nationwide Children's Hospital, Columbus, Ohio, USA; ^2^ Cancer Therapeutics Laboratory, Centre for Personalized Cancer Medicine, Discipline of Medicine, University of Adelaide, Adelaide, South Australia, Australia; ^3^ Division of Pediatric Hematology/Oncology/Bone Marrow Transplant at The Ohio State University, Columbus, Ohio, USA

**Keywords:** Ewing sarcoma, LSD1, EWS-FLI, epigenetics, methylation

## Abstract

Ewing sarcoma is an aggressive primary pediatric bone tumor, often diagnosed in adolescents and young adults. A pathognomonic reciprocal chromosomal translocation results in a fusion gene coding for a protein which derives its N-terminus from a FUS/EWS/TAF15 (FET) protein family member, commonly EWS, and C-terminus containing the DNA-binding domain of an ETS transcription factor, commonly FLI1. Nearly 85% of cases express the EWS-FLI protein which functions as a transcription factor and drives oncogenesis. As the primary genomic lesion and a protein which is not expressed in normal cells, disrupting EWS-FLI function is an attractive therapeutic strategy for Ewing sarcoma. However, transcription factors are notoriously difficult targets for the development of small molecules. Improved understanding of the oncogenic mechanisms employed by EWS-FLI to hijack normal cellular programming has uncovered potential novel approaches to pharmacologically block EWS-FLI function. In this review we examine targeting the chromatin regulatory enzymes recruited to conspire in oncogenesis with a focus on the histone lysine specific demethylase 1 (LSD1). LSD1 inhibitors are being aggressively investigated in acute myeloid leukemia and the results of early clinical trials will help inform the future use of LSD1 inhibitors in sarcoma. High LSD1 expression is observed in Ewing sarcoma patient samples and mechanistic and preclinical data suggest LSD1 inhibition globally disrupts the function of EWS-ETS proteins.

## INTRODUCTION

First described by James Ewing in 1921 as “diffuse endothelioma of bone,” Ewing sarcoma is the third most common malignant bone neoplasia diagnosed in children and adolescents [1, 2]. While the histogenesis of Ewing sarcoma remains enigmatic, 85% of cases are defined by the t(11;22)(q24;q12) chromosomal translocation, resulting in a pathognomonic chimeric fusion gene, *EWSR1-FLI1*, which encodes the EWS-FLI protein [3]. Treatment protocols continue to rely upon conventional multidisciplinary approaches coupling intensive chemotherapy with surgery and/or radiotherapy. The implementation of chemotherapy has achieved event-free survival rates nearing 75% for patients with localized disease, however those presenting with overt metastasis (seen in 20-30% of patients at diagnosis) or recurrent disease have poor outcomes, with under 30% disease-free survival [4, 5]. Furthermore, successful chemotherapeutic regimes are associated with significant cumulative and late toxicities [6]. Given the failure of systemic chemotherapy to improve durable remission rates for patients with metastatic disease, translation of novel therapeutic strategies to increase overall survival rates remains imperative.

Several challenges persist in the development of targeted therapies for Ewing sarcoma. Although advances in next generation sequencing have augmented our understanding of disease mechanisms driven by EWS-FLI, the cell-of-origin is unknown. Large-scale genomic sequencing efforts have demonstrated Ewing sarcoma possesses one of the lowest mutation rates amongst all cancers (0.15 mutations/Mb) [7, 8], yielding a paucity of pharmacologically actionable mutations. Indeed, Ewing sarcoma tumor samples showed recurrent, though low frequency, mutations only in the cohesin complex subunit *STAG2* (21.5%), the tumor suppressor *TP53* (6.2%) and homozygous deletion of the cyclin-dependent kinase inhibitor *CDKN2A* (13.8%) [7]. It appears possible Ewing sarcoma cells require large-scale epigenetic alteration to maintain malignant programming which disrupts normal developmental processes [9–15]. Notably, EWS-FLI blocks mesenchymal differentiation and promotes neuronal programs, which is in turn, dampened by EWSR1 and REST [13, 16]. Morphological studies suggest Ewing sarcoma cells strike a delicate balance between proliferative growth and metastatic capacity along the mesenchymal differentiation axis [17]. The transcription factor ZEB2 is critical to block expression of genes characteristic of an epithelial lineage [18]. Taken together, the oncogenic interplay of EWS-FLI with varied developmental pathways is marked by complexity. If Ewing sarcoma is to be placed within a Waddington landscape, perhaps it is best categorized as lost in the wilderness.

The importance of epigenomic misregulation in cancer and development of pharmacological tools to probe epigenetic mechanisms have advanced significantly in the past decade. However, the field faces technical hurdles in both collecting data and approaching the complexity in gathered data. Ewing sarcoma and other mutationally quiet pediatric malignancies have emerged as interesting model systems to further probe epigenetic aberrations conspiring in oncogenesis [7, 8, 19–21]. EWS-FLI expression affects the transcriptome, epigenome, and proteome to reprogram cells into a malignant developmental limbo [7, 8, 22–37]. Conversely, several studies suggest cellular context, both epigenetic and otherwise, influences the effects of EWS-FLI, as enforced expression in animal models leads to phenotypically variant tumors [38–40]. Moreover, expression of EWS-FLI in human pediatric mesenchymal stem cells failed to produce tumors in xenograft models, despite recapitulation of disease-specific transcriptomic and epigenomic phenotypes [31]. Rational design and implementation of improved therapeutic regimens requires more comprehensive understanding of disease mechanisms influenced by EWS-FLI and other FET/ETS fusions. Toward this end, recent work has described the epigenomic landscape of EWS-FLI in patient-derived cell lines and primary tumor samples [23, 29, 30, 35]. Additional lines of inquiry have further defined an important role for EWS-FLI in altering transcript splice selection [32, 33]. Notably, disruption of either epigenetic mechanisms or alternative splicing mechanisms delay tumor growth in xenograft models [22, 33].

Methylation is an important and subtle chemical modification which regulates chromatin status and is observed on both DNA and histones. Indeed, the significance of DNA methylation in both cancer initiation and progression has been appreciated for a number of years, resulting in the approval of two agents for the treatment of patients with myelodysplastic syndrome, azacitidine/Vidaza [41] (nucleoside analogue) and decitabine/Dacogen [42], (irreversible inhibitor of DNA methyltransferase enzymes DNMT1 and DNMT3). Histone methylation, a mechanism to modify chromatin structure, dynamically regulates cellular processes including transcription and genomic stability. Until a decade ago, histone methylation was considered an immutable modification, defining programs in concert with DNA methylation and other histone post-translational modification. However, the discovery of the first histone demethylase, lysine-specific demethylase 1 (LSD1) in 2004 [43], challenged this notion and proved lysine methylation is dynamically regulated. LSD1 (also known as *KDM1A, AOF2* and *BHC110*), is a flavin adenine dinucleotide (FAD) dependent amine oxidase with important epigenetic eraser function, specifically catalyzing oxidative demethylation of mono- and dimethyl-lysine at histone H3 lysines 4 and 9 (H3K4me1/2 and H3K9me1/2) [43], generating formaldehyde and hydrogen peroxide. In addition, LSD1 is reported to demethylate modified lysines on a myriad of non-histone proteins such as DNMT1 (residue K1096) [44], E2F1 (K185 residue) [45], MYPT1 (residue K442) [46], p53 (K370 residue) [47] and STAT3 (K140 residue) [48]. Importantly, LSD1 activity is highly context dependent. Several protein interaction partners are reported, including REST corepressor (CoREST) and MTA2, the nucleosome remodeling and deacetylase (NuRD) complex, and nuclear hormone receptors [49, 50]. As such, the epigenetic effects of LSD1 are implicated in diverse biologic processes pertinent to adipogenesis [51], chromosome segregation [52], cell proliferation [53], embryonic development [54], epithelial-mesenchymal transition (EMT) [55], hematopoiesis [56, 57], and regulation of stem cell pluripotency [58].

Owing to its high expression in several solid malignancies including breast [59–61], colorectal [62, 63], lung [62, 64], ovarian cancer [65], undifferentiated neuroblastoma [66], prostate carcinoma [67, 68], urothelial carcinoma [62], and sarcomas (Ewing, chondrosarcoma, osteosarcoma, rhabdomyosarcoma, and synovial) [69, 70], specific small molecule inhibitors of LSD1 have been aggressively pursued as potential therapeutics. Recently, our laboratory demonstrated EWS-FLI mediated transcriptional repression is facilitated through direct binding of a NuRD-LSD1 complex [71]. Furthermore, treatment of Ewing sarcoma cell lines with the potent and reversible LSD1 inhibitor HCI-2509, comprehensively reversed the transcriptional profiles driven by both EWS-FLI and EWS-ERG and significantly delayed tumorigenesis *in vivo* [22]. As such, this review will cover the rationale for LSD1 inhibition as a therapeutic strategy for Ewing sarcoma and the recent advances made by the scientific and pharmaceutical community to deliver potent LSD1 inhibitors.

## TARGETING HISTONE DEMETHYLATION IN CANCER

A variety of histone modifications, both written and erased by specific enzymes, are recognized by chromatin regulatory complexes to modulate target gene expression [72]. Methylation of histone H3 at lysine 4 (H3K4) and lysine 9 (H3K9) is linked to transcriptional activity at nearby genes [73]. In actively transcribing genes, H3K4me3 is strongly enriched at the transcription start site, with H3K4me2 and H3K4me1 peaks more broadly spread [74]. The methylation state of H3K4 is established through dynamic and coordinated activities of histone lysine methyltransferases, such as Set1/COMPASS family proteins, and histone lysine demethylases, such as FAD-dependent LSD1 and the JmjC domain containing JARID1 family [43, 75–77]. Notably, histone methylation is appreciated to function in precise cell- and tissue-specific manners [78, 79]. This may provide superior opportunities for therapeutic disruption in cancer, due to improved off-target profiles as compared to those observed with the use of histone deacetylase inhibitors [78–82]. LSD1 displays highly context dependent function, making it an attractive target to modulate epigenetic misregulation in cancer.

LSD1 demethylates H3K4 and H3K9 as well as non-histone protein substrates

The core structure of LSD1 comprises three domains, a small alpha-helical Swi3, RSC8, and MOIRA (SWIRM) domain and amine oxidase-like (AOL) domain form a closely packed structure and a protruding tower domain unique to LSD1 [49, 83]. The crystal structure of LSD1 shows similarity to other FAD-dependent oxidases and reveals a large substrate binding cavity to accommodate several residues of the N-terminal histone H3, positioning K4 for demethylation. This binding conformation is critical for the demethylation of mono- and di-methyl modifications, though the catalytic mechanism precludes activity against trimethylated lysine. In contrast to other SWIRM domain-containing proteins such as ADA2α and Swi3, the SWIRM domain of LSD1 lacks conserved DNA-binding residues, thus requiring other interaction partners to bind and demethylate native nucleosomes [83–85]. The tower domain directly protrudes from the catalytic center and binds LSD1-interacting proteins such as CoREST, MTA2, and HDAC1/2.

LSD1 was observed to demethylate the H3K9me1/2 marks in association with the androgen receptor, promoting target gene expression [50]. However, the mechanism by which LSD1 achieves this dual substrate specificity for both H3K4me1/2 and H3K9me1/2 marks was unclear until recently [86]. An alternatively spliced isoform of LSD1, LSD1+8a, can specifically demethylate H3K9me1/2, but not H3K4me1/2. This isoform was previously reported to be predominantly expressed in neuronal cell types and is involved in neuronal maturation [87]. Additional binding of the protein supervilin improved LSD1 catalytic activity toward H3K9me1/2, suggesting some interactions may favor specificity for one substrate over another. The expression of different LSD1 isoforms remains unexplored in Ewing sarcoma, but constitutes an important consideration in both cultured cells and patient-derived samples. Additionally, LSD2/KDM1B is a homolog of LSD1 (31% sequence similarity), lacking the tower domain while possessing additional zinc finger domains. LSD2 specifically acts within the gene bodies of target genes, displaying distinct localization of function as compared to LSD1, which acts primarily on promoter and enhancer regions [88, 89]. Expression of LSD2 in Ewing sarcoma likewise remains unexplored.

Apart from demethylating histone lysine residues, LSD1 has multiple non-histone substrates with diverse cellular functions, including p53, E2F1 and DNMT1. LSD1-mediated demethylation of p53 at K370me1/2 represses apoptotic activity, revealing a dynamic mode of p53 regulation [47]. Stabilization of E2F1 in p53-deficient tumor cells through demethylation at K185 inhibits DNA-damage induced cell death [45]. In an interesting link between the chromatin and DNA methylation machinery, LSD1 is critical for the maintenance of global DNA methylation patterns through regulation of DNMT1 stability during gastrulation in mouse embryos via demethylation at K1096 [90].

## ASSOCIATION OF LSD1 WITH MULTIPROTEIN COMPLEXES REGULATES FUNCTIONAL RESPONSES

The activity of LSD1 is predominantly in concert with specific chromatin regulatory complexes found in distinct cell types and several examples illustrate the functional specificity conferred through recruitment by specific factors. LSD1 commonly associates with CoREST and HDAC1/2 containing complexes for demethylation of nucleosomal substrates [91, 92]. In breast cancer cells, LSD1 interacts with MTA2 to forms an essential component of the NuRD complex containing MTA1/2/3 and HDAC1/2 to regulate key signaling mechanisms, including TGFβ signaling, involved in cell proliferation, metastasis and EMT [93]. During embryonic stem cell differentiation, LSD1 is required for the decommissioning and silencing of the pluripotency enhancers for normal differentiation, in cooperation with NuRD components [94]. Additionally, LSD1 is shown to play a crucial role in EMT *via* formation of a ternary Snail1-CoREST-LSD1 complex, in which LSD1 interacts with the SNAG domain of Snail1 to regulate target gene expression involved in the suppression of cell migration and invasion [95]. Other transcription factors which possess the SNAG domain, homologous to the N-terminus of histone H3, recruit LSD1 as well. During hematopoietic stem cell differentiation, the LSD1-CoREST complex is recruited by the SNAG domain of Gfi-1 to mediate repression of lineage-specific target genes [96]. The wide range of LSD1 interaction partners remains beyond the scope of this review. However, LSD1 is critical for execution of various differentiation programs including adipogenesis, skeletal muscle differentiation and pituitary organogenesis, highlighting the importance of histone demethylation in regulating cell-type specific gene expression patterns [51, 97, 98].

Although LSD1 regulates the expression of several downstream targets, the upstream regulators of LSD1 have not been extensively studied. miR-137 was shown to regulate the protein levels of LSD1 in neural cells through targeting the 3′UTR of LSD1, resulting in negative regulation of neural cell proliferation and increased neural differentiation [99]. miR-137 was also shown to be a tumor suppressor in neuroblastoma by downregulation of LSD1 [100]. Posttranslational control of LSD1 expression is dynamically achieved by ubiquitination. Stabilized levels of LSD1 by deubiquitinase USP28 conferred stem cell like properties to breast cancer cells [101]. Another study showed that an E3 ubiquitin ligase, Jade-2 (jade family PHD finger 2) can negatively regulate LSD1 in developing mouse cortical neurons and zebrafish embryos [102]. It remains to be explored whether these regulatory mechanisms are active in different cancers, including Ewing sarcoma.

## HIGH LSD1 EXPRESSION PROMOTES CELL PROLIFERATION AND METASTASIS IN CANCER

LSD1 is overexpressed in both solid and non-solid tumors such as breast, lung, colon, prostate, gastric cancers and acute myeloid leukemia (AML), playing significant roles in cell proliferation, cell migration and metastasis [59, 62, 67, 69, 103–106]. Functional downregulation of LSD1 expression or pharmacological inhibition significantly reduces tumor cell proliferation and metastatic progression in several malignancies *in vitro*. Although overexpression of LSD1 (mRNA and/or protein) is reported across numerous malignancies, few studies have evaluated whether LSD1 expression correlates with either cancer progression or overall survival. For those studies that have investigated the role of LSD1, limited patient cohort size and lack of clinical follow-up data has generally impeded the ability of studies to achieve statistical significance. In neuroblastoma [66], prostate [68] and ovarian [65] cancer, low LSD1 mRNA levels were predictive of event free survival. In breast cancer, LSD1 expression levels increased considerably during tumor progression from pre-invasive to invasive ductal breast carcinoma [60]. Conversely, in urothelial carcinoma, LSD1 expression levels were significantly high even in early grade (G1) tumors [62], implying that LSD1 is involved in tumor initiation for this malignancy. Wu et al., recently performed a meta-analysis to assess the association between LSD1 expression and overall survival in 1,149 cancer patients (hepatocellular carcinoma, esophageal, colon, breast, melanoma and tongue cancer). Analysis from nine studies, particularly enriched for Asian cohorts suggested that LSD1 overexpression was asso­ciated with poor overall survival, particularly for esophageal cancer patients (*P* = 0.000) [107].

Although expression of LSD1 has been evaluated in a large cohort of sarcomas, there is currently no evidence supporting the role of LSD1 in sarcoma tumor progression or disease-free survival. Immunohistochemical staining of a cohort of 468 sarcomas by Schildhaus et al., reported pronounced LSD1 expression specifically in highly malignant tumor groups including synovial sarcomas, rhabdomyosarcomas, desmoplastic small round cell tumors and malignant peripheral nerve sheath tumors (MPNST) [70]. A subsequent report by Bennani-Baiti confirmed these findings and extended the high expressing LSD1 sarcoma groups to Ewing sarcoma, chondrosarcoma and osteosarcoma [69], subtypes which were not investigated by Schildhaus et al. Although LSD1 expression in Ewing sarcoma was comparable to that of rhabdomyosarcoma, the most significant LSD1-expressing sarcoma subtype, no studies have hitherto to date examined the prognostic value of LSD1 expression in Ewing sarcoma.

## TRANSLATION OF LSD1 INHIBITORS

The well-defined active site cavity of LSD1 has enabled the development of numerous high-affinity and selective small-molecule inhibitors, reviewed extensively by Zheng et al., [108] and Mould et al., [109]. Unfortunately, numerous candidates failed to satisfy the stringent physicochemical and toxicological requirements for clinical development or were subsequently proven to possess poor LSD1 specificity profiles. Several peptide based LSD1 inhibitors (linear and cyclic) have also been generated [110–112]; however the clinical development pathway for peptide-based therapeutics remains uncertain.

As single-agent therapy increases the likelihood of the emergence of resistant cancer cell clones, the ability of LSD1 inhibitors to synergize with current treatment regimens will be imperative for their implementation into standard treatment protocols. The strongest evidence for potential combinatorial agents is for HDAC inhibitors. Co-treatment of glioblastoma cells with tranylcypromine and vorinostat led to a marked (6-fold) increase in caspase 3 activity [113]. In addition, treatment of primary AML blasts with the pan-HDAC inhibitor panobinostat significantly enhanced HCI-2509 induced apoptosis *in vitro* and significantly improved the median survival of mice, compared to treatment with HCI-2509 or panobinostat alone [114].

## PRECLINICAL EVALUATION OF LSD1 INHIBITORS IN SARCOMA

Previously prescribed as an anxiolytic and antidepressant to patients with anxiety or mood disorders [115], tranylcypromine (TCP) was the first monoamine oxidase (MAO) inhibitor identified [116]. TCP exerts its inhibitory activity by covalently binding to FAD, forming an tetracyclic adduct in the amine oxidase-like (AOL) domain binding pocket [116, 117]. In addition to MAOs, TCP is also able to inhibit the demethylase activity of LSD1 and LSD2, with a Ki of 242μM and 180μM, respectively [88, 118]. Treatment of neuroblastoma [66] and breast cancer [59] cell lines *in vitro* and *in vivo* with early derivatives of TCP resulted in significant growth inhibition. However, these inhibitory effects were only achieved at supraphysiologic concentrations of TCP, 20-30 fold higher than the enzymatic IC_50_ for LSD1. In the context of sarcomas, Bennani-Baiti et al., reported TCP inhibits the proliferation of Ewing, osteosarcoma, rhabdomyosarcoma and chrondrosarcoma cell lines, albeit in millimolar ranges which cannot be reasonably achieved in clinical settings [69]. Schildhaus, et al. also demonstrated growth inhibition of synovial sarcoma cell lines following treatment with TCP and clorgyline [70]. In consideration of TCP's adverse toxicity and poor potency/selectivity (IC_50_: 20.7μM, 0.2μM and 0.95μM; LSD1, MAO A and MAO B respectively), several analogues of tranylcypromine were synthesized with enhanced potency and target selectivity. This was achieved through the addition of bulky, branched side chains and the modi­fication of the phenyl group using crystal structures of the LSD1 substrate cavity for rational design [119, 120]. Pargyline was initially cited as a suicide inactivator of monoamine oxidases, blocking the demthylation of H3K9 by LSD1 during androgen-induced transcription [50]. However, two subsequent studies demonstrated that pargyline failed to inhibit LSD1 activity toward demethylation of H3K4 [118, 121].

**Figure 1:**
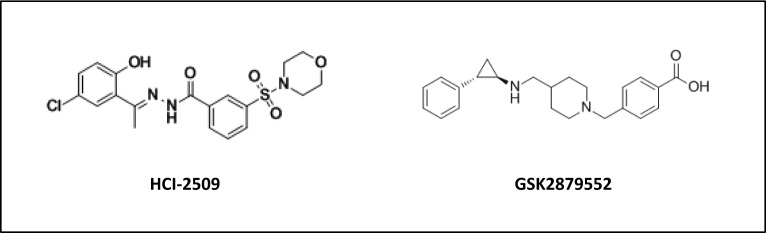
Chemical structure of HCI-2509 and GSK-2879552, reversible and irreversible inhibitors of LSD1 respectively

Recently, our laboratory investigated the therapeutic potential of a novel reversible and non-competitive LSD1 inhibitor (HCI-2509, Salarius Pharmaceuticals) for the treatment of Ewing sarcoma. HCI-2509, an N’-(1-phenylethylidene)-benzohydrazide small molecule (LSD1 IC_50_ 13nM; Figure 1), was originally identified through structure-based virtual screening [122]. Treatment of Ewing sarcoma cell lines with HCI-2509 treatment disrupted the global oncogenic activity of EWS-ETS fusions and induced apoptosis at physiologically relevant concentrations near 1μM, as will be discussed in detail below [22, 71]. In addition, cells expressing EWS-FLI were approximately 10-fold more sensitive to HCI-2509 treatment compared to cells with shRNA-mediated depletion of EWS-FLI, underscoring the specificity of LSD1 inhibition for Ewing sarcoma cells. Beyond Ewing sarcoma, HCI-2509 has also demonstrated single agent *in vitro* and *in vivo* efficacy in models of breast cancer [1 23], AML [114], poorly differentiated endometrial carcinoma [124] and castration resistant prostate cancer [125]. Clinical formulations of HCI-2509 analogues are currently being assessed and are expected to enter Phase I clinical testing within the near future.

## CLINICAL EVALUATION OF LSD1 INHIBITORS TO DATE

Although the development of specific LSD1 inhibitors is still in its infancy, three agents (tranylcypromine, GSK2879552 and ORY-100) are currently undergoing clinical evaluation, primarily in AML patients (Table 1). To date, the most promising pre-clinical data for LSD1 inhibition has come from mouse models of human AML (MLL-translocated). Foundational studies demonstrate the requirement of LSD1 for clonogenic and leukemia stem cell potential of MLL-AF9 AML cells and LSD1 action at genomic loci bound by MLL-AF9 to sustain expression of AML-associated oncogenic programs which prevent apoptosis and differentiation [126].

**Table 1:** Current LSD1 inhibitor trials

Compound	Study Title	Phase/Identifier	Sponsor	Stage
GSK2879552	Dose Escalation Study for GSK2879552 in Subjects With Relapsed/Refractory Acute Myeloid Leukemia	INCT02177812	GlaxoSmithKline	Recruiting
	Investigation of GSK2879552 in Subjects With Relapsed/Refractory Small Cell Lung Carcinoma	INCT02034123	GlaxoSmithKline	Recruiting
Tranylcypromine	Dose Escalation Study of Tranylcypromine (TCP) in Combination With ATRA (Tretinoin) for Adult Patients With Acute Myelogenous Leukemia and Myelodysplastic Syndromes	INCT02273102	University Of Miami	Not yet recruiting
	Pilot Trial of ATRA (Tretinoin) and TCP (Tranylcypromine) in Patients With Relapsed or Refractory Acute Myeloid Leukemia when no Intensive Treatment is Possible	I/IINCT02261779	Martin-Luther-Universität Halle-Wittenberg	Recruiting
ORY-100	Study of Human Pharmacokinetics and Safety of ORY-100 in relapsed or refractory acute leukemia	I/IIa2013-002447-29	Oryzon Genomics	Ongoing

ORY-1001 (structure undisclosed), a trans-2-phenylcyclopropylamine-based LSD1 inactivator (IC_50_ < 20nM) from Oryzon Genomics is reported to be 1,000 times more potent than TCP and highly selective over related enzymes, including LSD2. At sub-nanomolar concentrations (EC_50_< 1nM) ORY-1001 was shown to reduce leukaemic stem cell potential, colony formation and induce differentiation of AML cell lines [127]. GSK2879552 (GlaxoSmithKline) an N-substituted tranylcypromine derivative (Figure 1), is the first irreversible LSD1 inhibitor to be evaluated clinically in a solid tumor context (Small Cell Lung Carcinoma, SCLC). The recent screening of 165 cancer cell lines of varying histology by Mohammad et al., revealed that the anti-proliferative activity of GSK2879552 was largely restricted to SCLC and AML cell lines (EC_50_ 2-240nM), with genomic analyses revealing elevated MYC expression or amplification was correlated with resistance to GSK2879552, whereas global DNA hypomethylation was correlated with sensitivity [64]. In addition, treatment of AML cell lines promoted the expression of cell surface markers (CD11b and CD86) associated with a differentiated immunophenotype, and induced potent growth inhibition in patient derived bone marrow samples (EC_50_ 205nM) [128]. The results from these trials are eagerly awaited and will help further validate whether targeting the roles of LSD1 in cancer represents a tractable therapeutic option for patients.

## LSD1 MUTATIONAL STATUS IN SARCOMA

Accelerated cancer genome sequencing and high-throughput functional screen campaigns have significantly expanded our understanding of the abnormal biology and complex genetics of cancer cells. The resulting efforts have governed the discovery and development of targeted small molecules and laid the foundation for personalized medicine. Although molecularly targeted agents aimed specifically at drivers of pathogenesis have had some success, one mechanism of patient non-response can be attributed to pre-existing genetic mutation of the target gene itself. Fortunately, innate mutation of LSD1 is seldom observed. A recent search of cBioPortal revealed that mutation of LSD1 across numerous cancer subtypes is rare, with the highest mutation rates documented in urothelial bladder carcinoma (4/130 patients, 3.1%) [129], and medulloblastoma (1/37 patients, 2.7%) [130]. In congruence with this observation, mutation of LSD1 was not detected in either pediatric or adult Ewing sarcoma patients across five sequencing studies (*n* = 338 patients) (Table 2).

**Table 2:** Frequency of LSD1 mutation in Ewing sarcoma

Study	Sequencing platform	Frequency of LSD1 mutation in Ewing sarcoma patient cohort
Tirode, 2014^21^	WGS	0/112 (0%)
Crompton, 2014^8^	WES	0/92 (0%)
Brohl, 2014^7^	WGS	0/65 (0%)
Agelopoulos, 2015^154^	WES	0/50 (0%)
Huether, 2014^155^	WGS	0/19 (0%)

WES: Whole Exome Sequencing, WGS: Whole Genome Sequencing

## DRUGGING THE UNDRUGGABLE: UNDERSTANDING THE MECHANISMS OF ABERRANT EWS-ETS TRANSCRIPTION FACTORS

**Figure 2:**
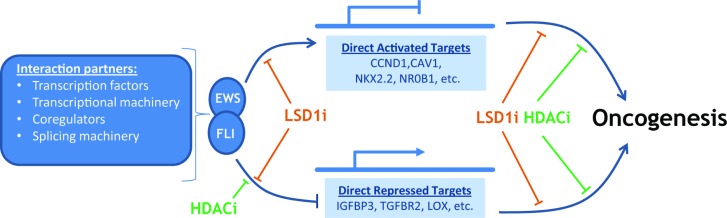
EWS-FLI interacts with multiple partners to cause gene specific activation and repression on the road to oncogenesis LSD1 inhibition (LSDi) negatively impacts direct transcriptional targets of EWS-FLI, in a manner distinct from HDAC inhibition (HDACi). Moreover, there is data to suggest additional roles for both LSD1 and HDACs in the downstream effects leading to oncogenesis, and these remain an area of active study.

The function of EWS-FLI, and other FET/ETS fusions characterizing Ewing sarcoma, is multifaceted and remains incompletely understood. What is clear is the related fusions sit atop a hierarchy of direct and indirect events which alter the composition of expressed genes, through disruption of both transcriptional and post-transcriptional processes in the cell. The downstream effects culminate in establishment and maintenance of an oncogenic phenotype as shown in Figure 2. Pharmacologically targeting protein-protein interactions and transcription factors remains a major challenge, but in Ewing sarcoma several persistent efforts are beginning to bear fruit. Here we discuss the mechanisms utilized by EWS-FLI that present tractable strategies for pharmacological blockade, specifically inhibition of chromatin modifications with a focus on LSD1.

## EWS-FLI FUNCTIONS AS AN ABERRANT TRANSCRIPTIONAL ACTIVATOR

Molecular mechanisms animating oncogenesis in Ewing sarcoma arise from the fusion of the N-terminal transcriptional modulation domain (NTD) of EWS with the C-terminal ETS-family DNA binding domain, which binds DNA elements possessing a core 5′-GGA(A/T)-3′ consensus motif [3]. The transcriptional activation induced by enforced EWS-FLI expression in NIH3T3 fibroblasts is sufficient for transformation [131]. Indeed, deregulation of the FLI transcription factor through N-terminal fusion with other strong activation domains, like VP16, shows transforming activity, highlighting an important role for transcriptional activation in Ewing sarcoma tumorigenesis [132]. Unlike full-length EWS, the NTD is shown to interact with the RNA polymerase II (PolII) complex subunit hsRPB7, nucleate the PolII complex, and recruit corroborating transcription factors, such as E2F3 and AP-1, and chromatin modifiers, like CBP/p300, to activate transcription [23, 30, 133–138].

Beyond the transcriptional deregulation resulting from imposition of the NTD in place of the regulatory N-terminal domain of FLI1, EWS-FLI shows emergent properties as an aberrant transcription factor at GGAA-microsatellites throughout the genome [26, 139, 140]. While several ETS-family members can bind GGAA repetitive elements *in vitro*, only EWS-FLI is able to both bind and activate transcription at nearby genes [139]. Recent deep sequencing studies interrogating the chromatin state at these GGAA microsatellites describe EWS-FLI recruitment of CBP/p300 to acetylate H3K27 and promote formation of enhancer elements at these loci [30, 35]. Several EWS-FLI activated targets indispensable for transformation, including NKX2.2, CAV1, GSTM4, and NR0B1 are regulated through EWS-FLI activity at nearby microsatellites, though none are currently candidates for therapeutic development [26, 30, 140–142].

## THE TRANSCRIPTIONAL HIERARCHY OF EWS-FLI INCLUDES GENE REPRESSION

Transcriptional profiling of patient-derived Ewing sarcoma cell lines using RNAi-mediated knockdown of EWS-FLI revealed a transcriptional signature which surprisingly showed more genes repressed by the fusion than activated. Several activated targets of EWS-FLI are, in fact, transcriptional repressors, including BCL11B, NKX2.2, NR0B1, and the long noncoding RNA EWSAT1 [27, 143–146]. Globally, the gene targets affected by these transcriptional repressors comprise only part of the EWS-FLI downregulated signature. Further investigation showed that EWS-FLI directly represses a subset of targets whose downregulation is required for tumorigenesis, including IGFBP3, TGFBR2, and LOX [71, 147, 148]. Moreover, EWS-FLI directly interacts with CHD4, MTA2, HDAC2, and HDAC3 in a NuRD-like complex at genes to enact transcriptional repression [71].

## TARGETING MECHANISMS OF GENE REGULATION

Both indirect repression, for example by NKX2.2, and direct repression depend upon the activity of histone deacetylases [71, 145, 146]. Thus, one potential therapeutic route blocks downstream epigenetic regulators which execute the repressive transcriptional program of EWS-FLI. Treatment with the HDAC inhibitor vorinostat derepresses gene targets of NKX2.2, BCL11B, and EWS-FLI and impairs cell viability and transformation in soft agar assays [71, 145, 146]. We further investigated whether targeting LSD1 might have similar effects in Ewing sarcoma models, as it commonly co-localizes with NuRD in the nucleus. Our initial investigation showed LSD1 inhibition with the small molecule HCI-2509 derepressed EWS-FLI-repressed targets and decreased cell viability in a manner comparable to HDAC inhibition [71]. More thorough transcriptional profiling in A673 and TTC-466 Ewing sarcoma cells intriguingly showed LSD1 inhibition flips both sides of the transcriptional profile for both EWS-FLI and EWS-ERG, respectively, upregulating repressed targets and vice versa [22] (Figure 2). This contrasts the HDAC inhibitor vorinostat, which diminished only EWS-FLI-driven gene repression and hints at roles for LSD1 in Ewing sarcoma biology beyond its documented corepressor activity, though the mechanisms of this activity remain unknown. For both HDAC and LSD1 inhibition, the effects on cell viability and transcription were mitigated in the context of RNAi-mediated EWS-FLI depletion, suggesting a disease-specific function for these enzymes [22, 71,]. Importantly, LSD1 inhibition with HCI-2509 showed single agent efficacy across multiple xenograft models for Ewing sarcoma [22].

While several groups have proposed a model for EWS-FLI activity whereby EWS-FLI binds microsatellite DNA and induces enhancer like features to promote gene activation at critical targets, the data to fully support any particular mechanistic model remain lacking [30, 35]. This complicates interpretation of the downregulation of activated targets by LSD1 inhibition considerably. However, LSD1 plays an important role in enhancer biology in embryonic stem cells (ESCs) and is required to repress the enhancers of pluripotency regulators during differentiation [94, 149, 150]. LSD1 is also reported to localize to enhancers of activated genes in ESCs and whether LSD1 is important for maintained gene activation or fine tuning of transcript levels in these contexts remains unclear [94]. Recent studies in more differentiated cell systems have shown cell-specific factors can toggle LSD1 substrate specificity between H3K4 and H3K9 at regulatory regions of target genes to promote either activating or repressive activity [151, 152]. In our hands, HCI-2509 showed more pronounced effects on global H3K9 methylation status in Ewing sarcoma, though the genomic implications of this result as well as a narrowed focus on H3K4 at regions of interest remain the work of continued studies.

## CONCLUSIONS AND FUTURE CHALLENGES

Exploiting the genetic addictions, vulnerabilities and esoteric dependencies of cancer cells has fueled a paradigm shift in conceiving new therapeutic strategies. Molecularly-targeted treatments informed by patient-specific characteristics are ascendant over non-specific cytotoxic therapies, where possible. Precision medicine aims to better address individuals' diseases while simultaneously reducing deleterious side effects. The quiet mutational landscape of Ewing sarcoma, coupled with documented overexpression of LSD1 and epigenetic misregulation, highlights the clinical potential of epigenetic inhibitors for the treatment of this aggressive malignancy. Indeed, small molecule blockade of DNA methyltransferase and histone deacetylases has proven epigenetic inhibitors are useful drug candidates. The low overall incidence of Ewing sarcoma 2.93 cases/1,000,000 [153], presents several challenges for the translation of novel agents into the clinic, and underscores the importance of global multi-center trial efforts to investigate the therapeutic potential of LSD1 inhibitors.

Although the use of LSD1 inhibitors for Ewing sarcoma shows promise, there are no reliable molecular biomarkers to predict either clinical activity or resistance to LSD1 therapy. Whether resistance will arise due to genetic mutation of LSD1 itself, activation of adaptive feedback loops or engagement of compensatory survival mechanisms outside the biological pathways targeted by LSD1 inhibitors remains unknown. It is widely accepted that single agent chemotherapy cannot constitute sole therapeutic intervention for Ewing sarcoma patients. As such, future work entails the evaluation of synergetic combinations which potentiate current Ewing sarcoma chemotherapeutic cassettes to provide a clear strategy for further LSD1 inhibitor clinical trials which mitigate drug resistance and achieve maximal effect. Although the initial signs are promising, results from ongoing clinical trials are eagerly anticipated. The rational coupling of mechanistic insight with translational science will ultimately determine whether these new epigenetic therapies comprise a meaningful addition to Ewing sarcoma treatment protocols.
